# The Maternal Capital Hypothesis: Giving Mothers Central Place in Evolutionary Perspectives on Developmental Plasticity and Health

**DOI:** 10.1002/ajhb.70084

**Published:** 2025-06-19

**Authors:** Jonathan C. K. Wells

**Affiliations:** ^1^ Childhood Nutrition Research Centre, Population Policy and Practice Department UCL Great Ormond Street Institute of Child Health London UK

**Keywords:** adaptation, developmental plasticity, health inequalities, intergenerational justice, maternal and child health

## Abstract

The “developmental origins of health and disease” paradigm has revolutionized biomedical research and raised new questions in the public domain. Not only individual disease risk, but also population health inequalities, may be profoundly shaped by experience early in life. The maternal capital hypothesis, published in 2010, is an evolutionary conceptual framework for understanding developmental plasticity on an intergenerational time‐scale. The central proposition is that societal adversities can become embodied in maternal phenotype, and hence undermine the health and life opportunities of their offspring. The offspring calibrates its early developmental trajectory to maternal phenotype, not to the external environment. The framework emphasizes societal stresses from which individual mothers cannot opt out, such as malnutrition, poverty, gender inequality, colonialism, racism, war, and interpersonal violence. Conversely, mothers with greater capital can better defend themselves against these stresses and buffer their offspring. In this commentary, I revisit why the hypothesis was developed and summarize how it has stimulated further work. I review evidence for the role of maternal phenotype in the intergenerational basis of health inequalities; theoretical issues that the hypothesis can help clarify; implications for policy and intergenerational justice; and experimental studies that show that promoting maternal capital can have health benefits for both mothers and offspring. There is no intention to blame mothers when arguing that maternal phenotype plays a unique role in intergenerational cycles of disadvantage. Rather, promoting maternal capital may not only improve maternal and child health, but also combat gender and racial inequality.

## 1 Introduction

As an anthropologist, I aim to bridge this academic field with public health nutrition using an evolutionary framework. If we can understand how societal dynamics impact our evolved physiological mechanisms, we may have a better idea of how to promote public health. Given that health outcomes show profound social patterning that persists across generations, this approach may also help explain and redress health inequalities. My empirical research focuses on low‐ and middle‐income countries, where child malnutrition, low education, and adolescent reproduction recur across generations in many populations, undermining both health and human capital.

After studying social and biological anthropology, I began a PhD in infant nutrition at the Dunn Nutrition Laboratory, Cambridge, United Kingdom. I learned to use stable isotope probes, sophisticated methods for exploring “what” and “how” questions by measuring body composition, breastmilk intake and energy expenditure. However, I was preoccupied with the “why” questions that characterize an evolutionary perspective.

I was fortunate to study in a group at the forefront of the emerging “developmental origins of health and disease” (DOHaD) field. While Barker and colleagues were publishing retrospective analyses, linking birthweight with adult noncommunicable disease (NCD) risk (Barker 1992), Lucas's group at the Dunn was conducting prospective research, randomizing babies to different diets at birth and studying child health outcomes (Lucas and Morley 1994). My PhD was observational, but I was very aware of the excitement and controversies surrounding DOHaD research.

The hypothesis that maternal nutrition shapes long‐term health of the offspring was investigated experimentally in animals (McMillen and Robinson 2005), but most human studies were observational or short‐term. The finding that individuals exposed to maternal famine in utero during the Dutch Hunger Winter had increased adult NCD risk offered unique evidence for the DOHaD hypothesis (Ravelli et al. 1998).

But why? Why should exposure to a relatively brief window of undernutrition during fetal life continue to shape health into old age? The “thrifty phenotype” hypothesis (Hales and Barker 1992) was arguably the first evolutionary perspective on the role of phenotypic plasticity in DOHaD. It proposed that the small baby had slowed growth of visceral organs relative to the brain, trading off long‐term NCD susceptibility against early survival.

Nutritional interventions during early life might therefore seem an obvious strategy to improve adult health. But paradoxically, supplementing pregnant mothers mostly produced little tangible benefit (Hawkesworth 2009; Kramer 2000).

What could an anthropologist contribute to the DOHaD field? Stimulated by the thrifty phenotype hypothesis, I identified two critical questions:

– What exactly was the environment shaping the fetus and infant?
– What was adaptive in the Darwinian sense about responding to this environment?

I first focused on Robert Trivers' concepts of parental investment and parent‐offspring conflict (Trivers 1972, 1974). I published a paper examining the roles of mother and fetus in the thrifty phenotype (Wells 2003), highlighting that both parties are under selective pressure to maximize their fitness. I argued that mothers with limited metabolic resources cannot afford large offspring, and that phenotypic plasticity was adaptive in allowing the offspring to match its development with the mother's capacity to invest in it. I emphasized that maternal physiology substantially buffers the offspring from external ecological stresses. In the Dutch famine, for example, maternal energy supply declined by 50%–60%, but offspring birthweight fell by only 9%.

This paper received some positive feedback, but soon there were several evolutionary perspectives on DOHaD. The predictive adaptive responses (PAR) hypothesis (Gluckman and Hanson 2004) proposed that low birthweight babies, exposed to maternal famine, develop characteristics such as central adiposity and insulin resistance in anticipation of famine persisting into adulthood. The intergenerational phenotypic inertia hypothesis (Kuzawa 2005) also considers that the fetus receives information predictive of future ecological conditions, but that this information has accumulated in maternal biology over a longer period, including through ancestral exposures. An alternative “developmental constraints” hypothesis assumes that poor environments restrict maternal investment, and that subsequent ill‐health is the consequence of “making the best of a bad start” (Jones 2005; Monaghan 2008).

I was skeptical that fetuses could gain reliable cues of their adult environment from transient exposure to maternal metabolism (Wells 2007). Even now, many evolutionary perspectives on DOHaD treat maternal phenotype as merely a proxy for the external environment (Malani et al. 2023), and yet this seemed to me to ignore the multiple ways in which mothers within any population might vary. For millions of years, humans and most primates have lived in “social food systems,” and these often involve forms of inequality and hierarchy. Understanding the mother's experience in society is crucial, for the mother *is* the only environment directly experienced by the fetus.

This was the focus of my paper on “maternal capital and the metabolic ghetto” (Wells 2010). Alongside the work of Trivers, Hales and Barker, two other conceptual papers stimulated me. Hill's comprehensive review of life history theory outlined how metabolic investment could be directed to competing ends (Hill 1993). That linked closely with the “evolutionary economics” framework of Kaplan et al., and the idea of phenotype representing different forms of embodied capital (Kaplan et al. 2003).

Integrating these ideas led me to the “maternal capital” hypothesis, aiming to shed new light on the intergenerational basis of developmental plasticity and its role in health inequalities. If my own paper has merit, it builds strongly on these seminal papers.

The maternal capital hypothesis emphasizes the following points:

– That the environment shaping the fetus, and to a large extent the infant, is not the external world, but rather different components of maternal capital.
– That these components go far beyond the mother's immediate nutritional status and relate to her own development, her reproductive strategy, and her position and capacity in society.
– That mothers can gain or lose different components of capital over different timescales and during different life‐course periods.
– That any adaptation by the offspring during the window of maternal physiological care is to maternal capital, a process subject to parent‐offspring conflict.
– That social stresses and inequalities impact maternal capital, and thereby shape the developmental trajectory and health of the offspring.

**FIGURE 1:**
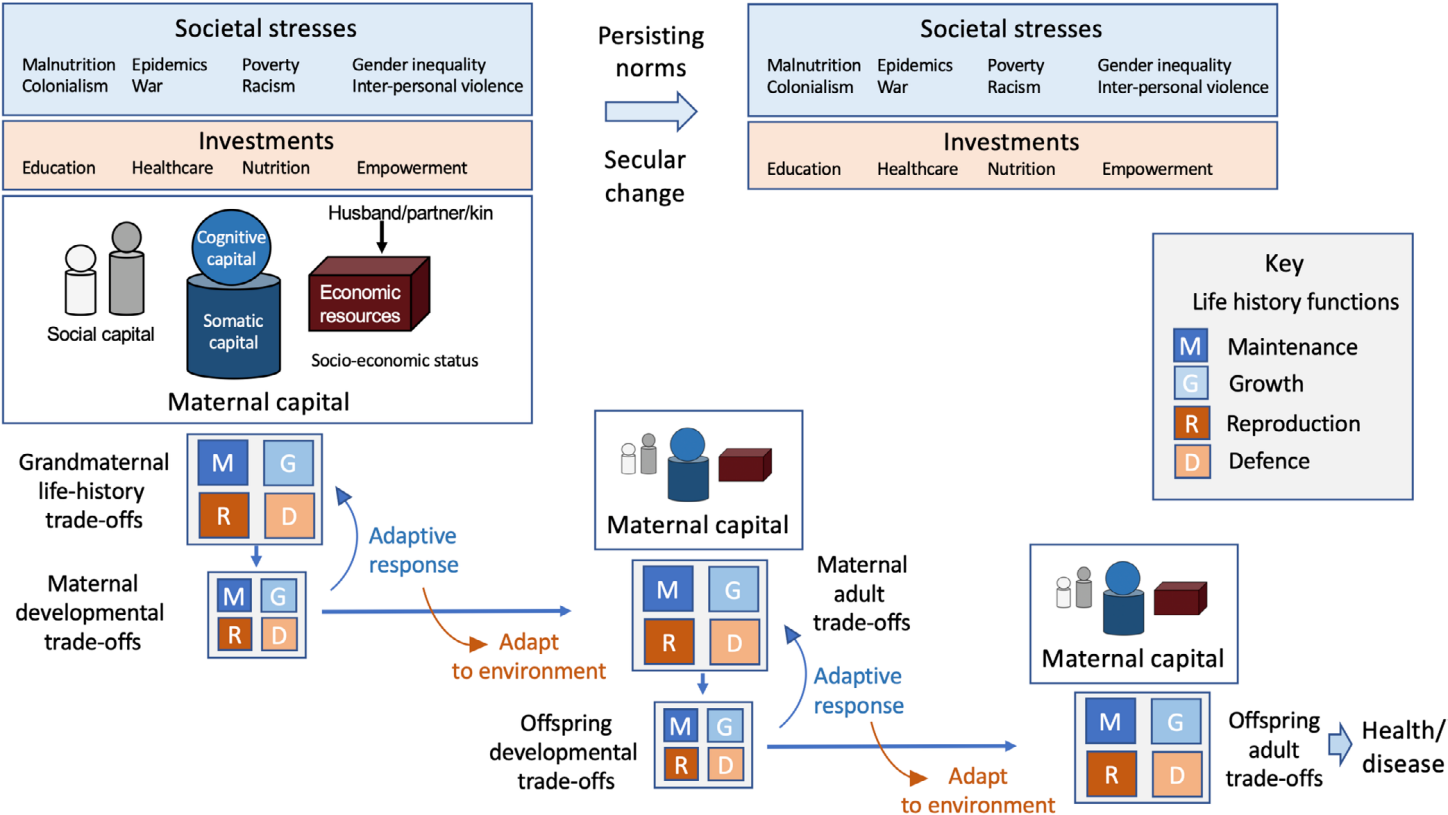
Schematic diagram of the maternal capital hypothesis. A range of societal stresses and investments shapes maternal capital, which influences life history trade‐offs between maintenance, growth, reproduction, and defense. Across three generations, from grandmother to grandchild, maternal capital in each generation first influences developmental trade‐offs in the offspring; these developmental trade‐offs then shape how each individual responds to further environmental exposures during childhood and adulthood. The offspring is exposed to a profile of maternal capital that was itself shaped during maternal development, and hence by grandmaternal capital. The interaction of capital transfer and ecological conditions cumulatively determines susceptibility to disease.

The risk of the DOHaD paradigm was that the mother might be treated like a cause of disease. I wanted an evolutionary conceptual framework that captured the opposite perspective: that while mothers can gain capital that helps them invest in their offspring, the societal adversities they face inflict biological penalties on them and, by depleting maternal capital, undermine the health of their offspring. Figure 1 provides a schematic diagram of the hypothesis, summarizing the points highlighted in this commentary.

Fifteen years later, it is gratifying to see that the maternal capital hypothesis has been cited often. Much use of the conceptual framework has been made by myself, my students, and colleagues, but also by others working across different academic disciplines. I will briefly summarize work that has drawn on the hypothesis in four related areas.

## 2 Life History Trade‐Offs: Health Inequalities and Cycles of Disadvantage

Life history theory assumes that all organisms are under selective pressure to invest their resources to maximize survival and reproductive fitness (Hill 1993). A key tenet is that in adverse conditions, growth and longevity are sacrificed to preserve defense and reproduction. Accordingly, exposure to low maternal capital could impose this trade‐off on the offspring.

I tested this hypothesis through my collaboration with the University of Pelotas, Brazil. Analyzing the 1993 birth cohort, we generated a composite index of maternal capital, based on mothers' weight, height, education, and household income. Mothers who were thin, short, uneducated and poor were categorized as having “low capital,” and these mothers gave birth to smaller daughters, who grew poorly in infancy and became adults with short height and low lean and fat mass, though also a more central fat distribution, benefitting immune function (Wells et al. 2019). Daughters of low‐capital mothers were more likely to have dropped out of school, to have reproduced by 18 years, and to have demonstrated risky behaviors such as smoking. Almost all outcomes showed dose–response associations, whereby the greater the maternal capital, the better the daughter's outcomes.

As predicted, therefore, daughters exposed to low maternal capital prioritized survival and early reproduction at a cost to growth, energy stores, and education. The findings for NCD risk were null, which may be because NCDs develop cumulatively through adult life. The low birth weight and central fat deposition of low capital daughters may still increase their susceptibility to NCDs in later life.

Overall, this study showed how low maternal capital contributes to intergenerational cycles of disadvantage, where the next generation may replicate many of the mother's characteristics. Benyshek drew on the maternal capital hypothesis when arguing that “the socio‐political marginalization, severe economic hardship, and nutritional inadequacy common to many Native American communities in the US during the reservation era” may help explain high rates of Type 2 diabetes among Native Americans (Benyshek 2013). Similar patterns are also seen in other species. For example, a study of olive baboons found that mothers who had experienced more adversity in their early life invested more in the care of their offspring compared to low‐adversity mothers, but still experienced greater levels of offspring mortality (Patterson et al. 2021).

However, such cycles can also be reversed if societal or ecological conditions improve. A Swiss study showed that over 60 years in the 20th century, average maternal height increased by ∼4 cm, and that birthweight of the offspring showed a similar upward trend following a 28‐year time‐lag (Floris et al. 2023).

Mechanistically, such intergenerational trends may involve different components of maternal capital. In prolonged poor conditions, populations may lose both weight and height across generations. To reverse this, I had suggested that mothers might first recover weight (energy stores), which could then enable their offspring to achieve better linear growth (Wells 2010). Consistent with that, analysis of data from 12 LMICs showed that mothers with overweight or obesity were less likely to have children who developed stunting (Wells et al. 2020). However, a caveat is that mothers with obesity are at risk of NCDs such as gestational diabetes, which may have adverse effects on offspring health (Farahvar et al. 2019). Thus, adiposity is a complex component of maternal capital, involving trade‐offs between maternal and offspring outcomes.

Alongside somatic capital, mothers also vary in components of social capital. For example, Alami et al., working in an Amazonian horticulturalist population, found that the children of politically influential women, indexed by verbal influence during community meetings, had better growth, nutritional status, and health (Alami et al. 2020). Marphatia et al. examined the reverse scenario, treating early women's marriage as a widespread practice that depletes maternal capital (Marphatia et al. 2017). They found that early marriage is associated with low education and undernutrition of women, and increased exposure to intimate partner violence. Subsequently, we explored in more detail the causes and consequences of early women's marriage in rural Nepal and India. We found that early marriage was associated with maternal thinness at the time of marriage (Wells et al. 2022a) and increased risk of preterm birth and malnutrition in the offspring (Miller et al. 2022; Wells et al. 2022b). Early marriage, along with other markers of depleted maternal capital, also predicted the child dropping out of school (Marphatia et al. 2019). In India, we highlighted another intergenerational cycle of disadvantage, whereby the daughters of early‐married mothers were more likely to marry early themselves and to replicate many of the associated adversities (Marphatia et al. 2025).

Women's empowerment and autonomy are components of maternal capital that may potentially benefit both maternal and child outcomes. However, studies have provided mixed evidence for offspring benefits. In Andhra Pradesh, India, greater maternal autonomy was associated with lower levels of stunting in children (Shroff et al. 2009). Conversely, in a large sample from the Democratic Republic of Congo, none of five dimensions of women's decision‐making power were associated with the risk of child malnutrition (McKenna et al. 2019). My interpretation is that, crucial as it is, we cannot expect women's empowerment on its own to fully negate the many ways in which societal stresses deplete maternal capital and undermine offspring health.

## 3 Theory: Adaptation, Calibration, and Buffering

The maternal capital hypothesis has been favorably reviewed among different evolutionary perspectives on DOHaD (Lea and Rosebaum 2020; Lu et al. 2019), though it remains very rare to see discussion of maternal fitness, as opposed to adaptive responses of the offspring. A key argument of the hypothesis is that the offspring calibrates its developmental trajectory to maternal phenotype, not to the external environment. To different degrees, all mothers constrain their reproductive investment in order to protect their own survival and future reproductive potential. Maternal investment depends not just on current resources, but also on the mother's own development and her reproductive scheduling.

In many settings, for example, firstborn offspring are born smaller than average, and then not only catch up but remain taller and heavier in later life, with implications for NCD risk (Kwok et al. 2016; Siervo et al. 2010; Wells et al. 2011). This developmental pattern may relate both to unique aspects of pregnancy physiology faced by firstborns, and also the lack of sibling competition in early postnatal life. This is a good example of a maternal effect that influences offspring development and adult health, despite having zero value for predicting future environmental quality (Wells 2016).

In a study of rhesus macaques, higher levels of glucocorticoids in the mother's milk were associated with trade‐offs in the offspring between behavior and growth rate, where a more nervous temperament spared more energy for growth. Moreover, higher milk glucocorticoids were more likely in low‐parity mothers or those in poorer condition. This study therefore suggests that mothers with lower maternal capital adaptively alter milk glucocorticoids to support the developmental trajectory of their offspring (Hinde et al. 2015).

More broadly, Berghanel et al. examined the effects of maternal stress during pregnancy on offspring development across mammals. They hypothesized that stressed mothers would reduce fetal investment, leading to the offspring recalibrating its developmental trajectory to mature faster after weaning. They supported the hypothesis, but only if the stress occurred early in pregnancy, and concluded that such mechanisms allow both the mother and offspring to make the best of the situation (Berghanel et al. 2016). Human studies likewise show that maternal stress impacts the offspring through hormonal mechanisms. In New Zealand, for example, mothers exposed to ethnic discrimination or economic deprivation had elevated evening cortisol levels, which in turn were associated with an elevated cortisol response of the infant to vaccination (Thayer and Kuzawa 2014, 2015).

All of these studies demonstrate the calibration of offspring early development to components of maternal phenotype that are variable within the population, inconsistent with the notion that maternal phenotype simply signals the quality of the future environment.

Moreover, part of the fitness value of maternal capital is precisely that it conceals the “true” state of the environment. In my early work I emphasized that maternal physiology smoothes over short‐term ecological perturbations and buffers the offspring, reducing its sensitivity to transient stresses (Wells 2003). Thayer et al. developed this further in the “nutritional buffering” hypothesis, exploring variability in the maternal regulation of different types of nutrient (Thayer et al. 2020).

Maternal hormones and nutrients thus act primarily as signals of maternal phenotype, rather than the external environment. Using mathematical models, Kuijper and Johnstone showed that under conditions of parent‐offspring conflict, a partial or total breakdown of ecological information transfer from mother to offspring is expected (Kuijper and Johnstone 2018). I also developed a simulation model with Johnstone of a fluctuating environment, based on rainfall data from India. The model showed that maternal effects effectively damp exposure of the fetus to external ecological volatility and maintain birth weight in a healthy range, but do not predict ecological conditions later in life (Wells and Johnstone 2017).

Contrasting with volatile ecological conditions, some maternal signals have particular value for the offspring. There is one component of maternal capital that is sensitive to ecological stress during the mother's own development, and which has profound implications for both mother and offspring, even if the stress never recurs. Poor growth of the mother reduces the dimensions of the birth canal and increases the risk of obstructed labor (Wells 2017), which can cause injury or death to both parties. Paradoxically, maternal nutritional intake during pregnancy may be less important in DOHaD than is widely assumed. Rather, both genetic and plastic mechanisms allow the fetus to align its growth with maternal pelvic dimensions, with implications for both birthweight and gestation length, and hence adult NCD risk (Wells et al. 2024). These adaptive mechanisms help explain why the dose–response association of birthweight and NCD risk runs across almost the whole birth weight range, and why maternal macronutrient supplementation in pregnancy only increases birthweight in chronically undernourished mothers. However, the regulatory mechanisms seem to be overwhelmed in mothers with elevated adiposity, increasing the risk of childbirth complications and C‐section (Wells 2017).

Finally, the maternal capital hypothesis assumes that mothers as well as offspring are under selective pressure to maximize their fitness. An intriguing perspective on this was provided by a study in Merida, Mexico. Mothers receiving instrumental support from their own mother had less favorable attitudes to breastfeeding than mothers lacking this support and breastfed their infant for less time (Vazquez‐Vazquez et al. 2022). It is possible that the grandmothers were acting more in the mother's interest.

## 4 Colonialism and Intergenerational Justice

The maternal capital hypothesis is a framework for understanding how societal pressures that adversely affect women become embodied and undermine the health and life opportunities of both mothers and their offspring. Health inequalities are evident across as well as within countries, and NCDs are rapidly increasing in LMICs through mechanisms that encompass the biological legacy of colonialism.

To emphasize the role of power relations in embodiment, I coined the term “metabolic ghetto,” drawing on an account of how urban ghettos were imposed on medieval Jewish populations (Wirth 1928). These ghettos had contrasting elements of incarceration, protection and vulnerability, and all of these characteristics could be reproduced when populations migrated to new settings. This scenario seemed to me to have substantial resonance with maternal metabolism—which might simultaneously buffer the offspring from external threats, but also expose it to past or current stresses impacting the mother. I wanted to focus on the stresses that emanate from society at large, from which individual mothers cannot “opt out.” Examples include malnutrition in all its forms, disease epidemics, poverty, gender inequality, colonialism, racism, war, and interpersonal violence. Few of these stresses had received attention in the developmental constraints or PAR literature, though all impact maternal biology.

In hindsight, while the term “ghetto” helped clarify my own thinking, it may have challenged readers. It can have different meaning in different contexts and has a range of historical connotations. Nevertheless, philosophers of biology such as Meloni have recognized the role this framework can play in helping dissolve the artificial boundary between the biological and the social (Meloni 2019). Meloni noted that the concept of maternal capital “crosses and undermines any possible organic/cultural or biological/social boundary we might draw,” and that this helps understand how “political oppression and economic marginalization … become embedded and transmitted to future generations” (Meloni 2016).

Thomson and Goldblatt referenced my discussion of colonial metabolic legacies in their work emphasizing the role of embodiment in perpetuating inequalities across generations (Thomson and Goldblatt 2025). This may interact with contemporary racisms, which continue to deplete maternal capital in minoritized populations (Selvarajah et al. 2022). Devakumar et al. reviewed the intergenerational consequences of war (Devakumar et al. 2014), while Gowland et al. analyzed the skeletal remains of children from 19th‐century England and revealed the intergenerational consequences of child labor (Gowland et al. 2023).

Several academics have invoked the maternal capital hypothesis in the context of intergenerational justice. Harris and Nisbett re‐examined the UNICEF framework for malnutrition to develop our understanding of the “basic determinants,” acknowledging that it is through maternal exposure to these factors that malnutrition recurs across generations (Harris and Nisbett 2021). Nisbett et al. highlighted “unfairness, injustice and exclusion as the engine of nutrition inequity across place, time and generations,” again noting the intergenerational dynamics (Nisbett et al. 2022).

There is no intention to “blame mothers” when arguing that women's phenotype plays a unique role in intergenerational cycles of disadvantage. This is particularly relevant when we consider that another way in which society systematically depletes maternal capital is through gender inequality. Marphatia et al. showed that, independent of national wealth, countries with worse levels of gender inequality also tend to have higher prevalences of low birthweight, child undernutrition, and under‐5 year mortality (Marphatia et al. 2016). In Nepal, moreover, mothers with low capital were more likely to have daughters than sons, for several underlying reasons. Compared to boys, therefore, girls in this setting are inherently born into households with lower capacity to invest in them, indicating a prenatal origin of gender inequality (Marphatia et al. 2022a).

## 5 Policy Implications

The maternal capital hypothesis has a number of implications for policy. Gender inequality permeates many areas of society, impacting education, political agency, employment, health, and reproduction. Since every human passes through the developmental niche of maternal phenotype, policies that promote maternal capital can benefit both mothers and all members of the next generation. Because maternal capital has many complementary constituent elements, such policies should be multisectoral. This requires greater integration of the organizations and ministries that target specific outcomes and sustained long‐term investment so that their combined efforts are more effective. Such policies can play a key role in redressing health inequalities associated with discrimination and marginalization.

Crucially, the full benefits of promoting maternal capital for subsequent generations will take time to manifest. Implementing such policies will require strong political will and the willingness to accept that those initiating the effort may not receive immediate recognition. However, successful policies have the potential to help redress past injustices and confer stable and long‐lasting benefits.

## 6 Experimental Research: Capital Transfer

The ideal way to test the maternal capital hypothesis experimentally is to consider capital transfer. Nutritional supplementation could potentially benefit both mother and offspring, but the results are often disappointing (Devakumar et al. 2016; Kramer 2000), and health outcomes are often only measured in the children. The ideal intervention would explicitly benefit mothers, while also passing benefits to the offspring: the win–win scenario.

Unlike many DOHaD researchers, my empirical focus has always been infant rather than fetal nutrition. For over 20 years I have co‐led a research group with Mary Fewtrell, studying lactation and growth in different settings. Recently, our PhD students have become fascinated by the idea that relaxation therapy could improve maternal mental health and breastfeeding.

Being responsible for an infant carries its own stresses. Societies typically scrutinize reproduction closely, imposing unique expectations on mothers. In Ethiopia, for example, pregnant women reported greater perceived stress and lower resilience compared to nonpregnant women (Abera et al. 2023). The World Health Organization advocates exclusive breastfeeding for the first 6 months after birth. This places not only physical but also psychological demands on mothers. Breastmilk consumption is invisible, and babies cry to express their hunger. Many mothers worry that they cannot produce enough milk (Huang et al. 2022), and this may be one reason why rates of exclusive breastfeeding remain low (Global Nutrition Target 2024).

Breastfeeding is not an inert transfer process, rather it is subject to maternal‐offspring conflict (Trivers 1974), a tug‐of‐war over valuable metabolic resources. Indeed, because breastfed infants have to work physically harder than formula‐fed infants to obtain their milk, they may be more fractious (Lauzon‐Guillain et al. 2012). Fewtrell et al. described the role of diverse signals between mother and offspring, representing “negotiation” in the tug‐of‐war (Fewtrell et al. 2020).

Our student Husna Mohd Shukri hypothesized that relaxation therapy would rebalance this contest. She conducted a randomized trial in Malaysia, allocating healthy first‐time mothers to relaxation therapy (listening to an audio‐cassette) while breastfeeding, or to have standard breastfeeding support. The intervention mothers reported lower stress and produced more breastmilk, with greater carbohydrate and lower cortisol context. Their infants slept longer and showed greater weight gain than those in the control group (Mohd Shukri et al. 2019).

These results were obtained without providing any extra food to the mother; instead, the intervention altered maternal life history trade‐offs, lowering energy allocation to the stress response and thus releasing energy for reproduction. Two other students conducted similar trials in mothers of late preterm or early‐term infants, who may be more difficult to feed. The results were unanimous: relaxation therapy reduces maternal stress markers and promotes infant growth (Dib et al. 2022; Yu et al. 2023). Moreover, the intervention seems most effective in the mothers with the lowest capital at baseline, indicating the potential of this approach for reducing health inequalities (Dib et al. 2023).

## 7 Conclusion

I hope that the maternal capital hypothesis has helped break new ground as intended, by helping understand the intergenerational aspect of the developmental origins of health inequalities and identify potential solutions. Exposure to low maternal capital increases susceptibility to poorer outcomes, but infants and children also show resilience, and interventions through the life course can lead to better outcomes. The evolutionary framework is not exclusive to humans and can be used by zoologists exploring mother‐offspring dynamics.

In 2016 I published a book, “The metabolic ghetto,” which developed a more comprehensive perspective on the societal drivers of health inequalities, and how NCDS arise through the interaction of evolved mechanisms of developmental plasticity with diverse forms of power relations (Wells 2016). While most DOHaD research examines solutions at the individual level, policy responses are urgently needed to mitigate societal stresses.

Clearly, this hypothesis says little about paternal biology, though it does acknowledge paternal capital, often accessed by women through marriage or other partnerships (Marphatia et al. 2022b). This is not to ignore mechanisms through which fathers can transmit biological or behavioral effects to offspring (Crean and Bonduriansky 2014). Also, neither pregnancy nor lactation is exclusive to mothers (Obedin‐Maliver and Makadon 2016; Slome 1956), and allomothers' capital merits attention. Rather, I aimed to focus on the twin facts that mothers play the primary physiological role in parental investment and also typically carry the primary burden of unpaid care. Policies and interventions that promote maternal capital may help address gender inequality as well as maternal and child health.

## Ethics Statement

The author has nothing to report.

## Conflicts of Interest

The author declares no conflicts of interest.

## Data Availability

Data sharing is not applicable to this article as no datasets were generated or analysed during the current study.
